# Diagnostic immune-related markers for diabetic kidney disease: a bioinformatics and machine learning approach

**DOI:** 10.1080/0886022X.2025.2525467

**Published:** 2025-07-10

**Authors:** Ying Wang, Xinyuan Zhou, Yuxin Jiang, Ling Jiang, Li Gao, Xueqi Liu, Xiaoxia Wang, Chenyu Sun, Yonggui Wu

**Affiliations:** a Department of Nephropathy, First Affiliated Hospital of Anhui Medical University, Hefei, P.R. China; b Department of Biostatistics of Epidemiology, School of Public Health, Anhui Medical University, Hefei, P.R. China; c AMITA Health Saint Joseph Hospital Chicago, Chicago, IL, USA

**Keywords:** Diabetic kidney disease, bioinformatics analysis, immune infiltration, ALB, FOS, S100A9

## Abstract

**Objective:**

Diabetic kidney disease (DKD) is a leading cause of chronic kidney disease, with chronic inflammation driving its progression. This study aimed to identify immune-related diagnostic biomarkers for DKD and explore their association with immune cell infiltration.

**Methods:**

Three glomerular transcriptomic datasets (53 DKD, 36 controls) were analyzed via batch-corrected differential expression analysis to screen immune-related differentially expressed genes (DEGs). Machine learning algorithms (least absolute shrinkage and selection operator, support vector machine - recursive feature elimination) prioritized biomarkers, validated by RT-PCR in db/db mice. Immune infiltration was assessed via CIBERSORT and EPIC.

**Results:**

Thirteen immune DEGs were identified, enriched in cytokine signaling and leukocyte chemotaxis. Three biomarkers (albumin (ALB), AP - 1 transcription factor subunit (FOS), and S100 calcium binding protein A9) showed strong correlations with T cell, natural killer cell, and macrophage infiltration, validated by RT-PCR (*p* < 0.001). Protein-protein interactionnetwork analysis identified ALB and FOS as hub genes (ROC Area Under the Curve: 0.803, 0.795), linking immune dysregulation to glomerular injury.

**Conclusion:**

ALB and FOS serve as novel immunogenetic biomarkers for DKD, highlighting chronic inflammation as a key driver. This framework supports precision immunomodulation, though clinical validation in larger cohorts is needed.

## Introduction

The prevalence of diabetes and chronic kidney disease (CKD) is rising rapidly, resulting in an increase in the number of new diagnoses of diabetic kidney disease (DKD) and end-stage kidney disease (ESKD) worldwide. Although the proportion of new ESKD cases attributed to DKD varies by country, DKD accounts for nearly half of the primary causes of ESKD in many nations, including Singapore and the United States [1,2]. The pathogenesis of DKD is complex and multifactorial. Despite the widespread use of treatment strategies such as glucose regulation and renin-angiotensin system inhibitors, inadequate therapeutic options for diabetes and its complications remain a significant challenge, affecting approximately 77% of patients in low- to middle-income countries [2]. Effective treatment strategies are urgently needed to slow the progression of DKD, however, no novel direct therapies have been introduced in the past two decades. While emerging therapies such as Sodium-Glucose Cotransporter 2 Inhibitors (SGLT2i), Glucagon-like Peptide-1 Receptor Agonists (GLP-1 RA), and mineralocorticoid receptor antagonists (MRA) have shown renoprotective effects [3], their mechanisms remain partially understood, and a significant proportion of patients still progress to ESKD. This underscores the need for novel biomarkers that can stratify disease risk and guide targeted interventions. Substantial experimental evidence suggests that chronic inflammation plays a critical role in the development of diabetes and its associated vascular complications, including DKD. Preclinical studies have identified several novel molecules linked to the innate immune system that show promise in reducing albuminuria and/or proteinuria. Recently, the role of chronic inflammation in the pathogenesis of DKD has gained significant attention in both clinical and experimental research [4]. Therefore, this study aims to investigate potential immune-related biomarkers and examine their involvement in the persistent inflammatory response associated with DKD, offering new insights into its pathophysiology and identifying potential therapeutic targets.

Growing evidence suggests that low-grade chronic renal inflammation contributes significantly to the pathogenesis and progression of DKD. Elevated levels of high-sensitivity C-reactive protein (hs-CRP), an inflammatory marker, have been associated with this condition [5–8]. Immune cell infiltration is a well-known characteristic of DKD [9]. A single-nucleus RNA sequencing (snRNA-seq) study on cryopreserved human diabetic kidney samples revealed markedly increased immune cell infiltration in diabetic samples. These infiltrating immune cells contribute to the production of kidney risk inflammatory signature (KRIS) markers, a group of 17 circulating plasma inflammatory proteins that predict DKD progression [10,11]. Numerous susceptibility genes associated with DKD have been identified, many significantly enriched in infiltrating immune cells. These genes may help pinpoint DKD signaling pathways and potential early biomarkers. Collectively, this data suggests that inflammation may drive late diabetic complications, and immunotherapy could offer renoprotective effects for DKD patients. Several anti-inflammatory molecules targeting chemokines, transcription factors, adhesion molecules, and kinases have shown promising results in preclinical and early-phase clinical studies [12]. Nevertheless, due to the immune dysfunction associated with both diabetes and advanced DKD, these nonspecific anti-inflammatory treatments may increase the risk of infection in DKD patients, limiting their clinical application. Although new treatments like SGLT2i, GLP − 1 RA, and MRA have been developed and have shown significant protective effects on DKD, including better diabetes control, slower renal function decline, and reduced cardiovascular risks, the overall treatment situation for DKD, especially in low- to middle-income countries, is still far from ideal. Our study is dedicated to exploring immune-related biomarkers for DKD. Despite the progress brought by new therapies, there is still a need for more optimal treatment solutions. Thus, further research on their benefits in DKD patients is essential. It helps in understanding the efficacy and safety of these treatments and developing more comprehensive strategies, thereby addressing unmet medical needs and improving the prognosis of patients. Moreover, a thorough understanding of the role of innate immunity in triggering local low-grade inflammation and promoting pathophysiological changes in DKD may facilitate the development of more specific, novel, and less toxic anti-inflammatory therapies for this condition.

To conduct a comprehensive analysis, we selected three datasets from the Gene Expression Omnibus (GEO) database. For GSE30528, it includes 9 diabetic patient samples and 13 normal samples, all being renal tubular cell samples. The patient samples are at different clinical stages, which is ideal for exploring the impact of diabetes on the kidneys and gene expression changes during DKD development. Analyzing this dataset *via* the Affymetrix GPL571 platform can help identify immune-related differentially expressed genes. GSE1009 provides gene expression data at the individual level, with 3 diabetic and 3 normal samples obtained from kidney tissues. This dataset, based on the Affymetrix GPL8300 platform, is crucial for exploring diabetes and complication biomarkers and can validate gene markers from larger datasets. GSE96804, a relatively large dataset, contains 41 diabetic patient samples and 20 normal samples. Focusing on diabetic gene expression with various clinical parameters, and using the Affymetrix GPL17586 platform, allows in-depth gene analysis, especially regarding the immune response in diabetes progression.

Bioinformatics analysis is a computational method used to capture and interpret biological data. In recent decades, next-generation sequencing (NGS) technology has become a powerful tool, revealing vast amounts of biological information related to both tumorigenesis and non-neoplastic diseases. Large-scale gene expression data for various diseases are now publicly available. Several studies using microarray analyses have identified differentially expressed genes (DEGs) and biological pathways associated with DKD [13,14]. However, immune-related DEGs in DKD have not been precisely identified, and immune infiltration levels have not been thoroughly investigated. Variations in microarray platforms, statistical methods, and small sample sizes may contribute to conflicting results across studies. Therefore, comprehensive analyses, including immune infiltration assessments, are essential to identify more robust and novel therapeutic targets for DKD.

The current study aimed to identify key immune-related gene biomarkers and their association with immune cell infiltration in the glomeruli of DKD. Datasets from DKD patients and healthy controls were analyzed using the GEO database through comprehensive bioinformatics methods. Alternative computational algorithms, specifically the CIBERSORT [15] and EPIC [16] methods, were employed for immune cell quantification to define and compare the immune cell landscape in the glomeruli of DKD patients and normal controls, revealing distinct immune phenotypes for molecular subclasses. We then assessed the diagnostic value of the key potential gene biomarkers using receiver operating characteristic (ROC) curves. Additionally, reverse transcription polymerase chain reaction (RT-PCR) was conducted to verify the expression levels of the identified gene biomarkers in kidney tissue from type 2 diabetic db/db mice. This study established the first comprehensive network of genes genetically associated with immune infiltration and responses in DKD, providing a valuable foundation for understanding the molecular mechanisms underlying immune infiltration in DKD.

## Methods

### GEO dataset collection and study design

**Figure 1.**
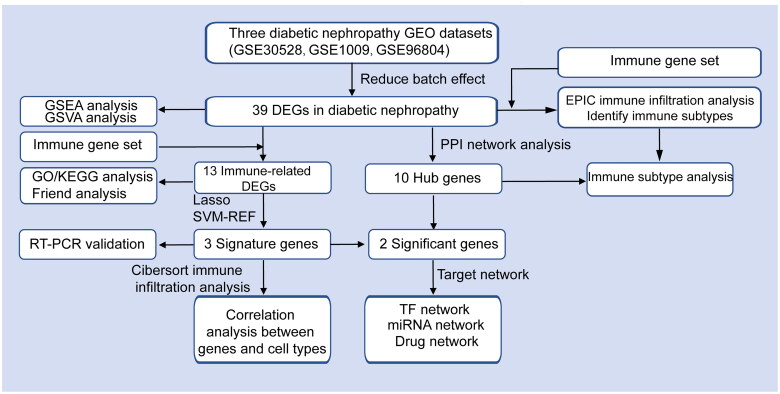
Flow diagram of the experiment and analyses in this study.

The NCBI GEO database [17] is a publicly available repository for genomics data, which archives and distributes microarray, next-generation sequencing, and other high-throughput functional genomic datasets. Three gene expression profiles were downloaded: GSE30528^18^ (DKD = 9, normal = 13), GSE1009 [18] (DKD = 3, normal = 3), and GSE96804 [19] (DKD = 41, normal = 20) from the GEO database. These datasets were used to screen and verify hub genes involved in the glomerulus. The species studied was Homo sapiens. The datasets were derived from the Affymetrix Human Genome platform, with platforms GPL571, GPL8300, and GPL17586, respectively (Table 1). The Human Phenotype Ontology (HPO), C2 (C2.all.v7.2.symbols.gmt) online pathway database, and biomedical literature-related gene sets were downloaded from the MSigDB [20] (https://www.gsea-msigdb.org/gsea/msigdb). The immune function-related gene set was retrieved from Immport Database [21] (https://www.immport.org/home). Data analyses were performed using R software (version 3.4.0; https://www.r-project.org/) and Bioconductor packages (http://www.bioconductor.org/). The workflow overview is shown in Figure 1.

**Table 1.** The information of the three gene expression profiles GSE30528, GSE1009, and GSE96804.

Tissue	Dataset ID	GPL ID	No. of samples	Dataset type	Organism
Human glomeruli	GSE30528	GPL571	9 DKD 13 control	Microarray	Homo sapiens
Human glomeruli	GSE1009	GPL8300	3 DKD 3 control	Microarray	Homo sapiens
Human glomeruli	GSE96804	GPL17586	41 DKD 20 control	Microarray	Homo sapiens

GPL ID, The GEO Platform (GPL) identifier.

### Quality control and data standardization

First, the Robust Multi-Array (RMA) function in the ‘affy’ [22] R package was used to standardize the gene expression matrix. The gene expression values were log2-transformed and organized into information tables. The Array Quality Metrics [23] tool was applied to assess chip data quality by performing principal component analysis (PCA) and generating PCA plots for each dataset. Considering that different microarrays might come from different research conditions, which could lead to batch effects, we extracted the overlapping genes from the three datasets and merged them. Then, the ‘combat’ function in the ‘sva’ [24] R package was used to eliminate batch effects. After obtaining the expression profiles (DKD = 53, normal = 36), the quality of the data was evaluated both before and after batch correction, with box plots illustrating the differences.

### Identification of immune-related DEGs

DEGs in the three datasets, after batch correction, were identified using the ‘limma’ [25] R package. Statistical ­significance was defined by an adjusted *p* value of <0.05 and a log2|FC| > 1. The identified DEGs were visualized using Volcano plots generated with the ‘ggplot’ R package and heatmaps generated with the ‘heatmap’ R package.

### Immune subtype analysis

To identify immune subtypes, consensus clustering was used as a robust quantitative method to determine the number of clusters and their components. The optimal number of clusters was inferred by selecting an appropriate K value. Consistency clustering verified the clustering’s rationality using repeated sampling to evaluate stability. The ‘Consensus Cluster Plus’ [26] R package was employed to intersect the profiles of the batch-corrected datasets with the Immport immune-related database (https://www.immport.org/home). Consensus clustering was then performed on the immune-related overlapping gene profiles. Heatmaps, Cumulative Distribution Function (CDF) maps, and Delta Area maps were used to identify the optimal number of clusters. Principal component analysis (PCA) was performed based on the selected number of clusters, followed by the creation of a PCA plot.

### EPIC immune infiltration analysis

To analyze the immune characteristics of the samples and identify potential features, the EPIC immune infiltration analysis was applied to the batch-corrected profiles. The EPIC ^16^database (https://gfellerlab.shinyapps.io/EPIC_1-1/) was used to determine the degree of immune cell infiltration in the samples based on deconvolution algorithms. Stacked bar charts were created to visualize the distribution of immune cell types. Correlation analyses between immune cell types were performed, and correlation heatmaps were generated. Finally, box plots were used to compare immune cell infiltration between DKD patients and normal controls based on predefined grouping criteria.

### Gene and gene set enrichment analysis (GSEA)

The GSEA [27] algorithm evaluates the correlation of identified genes with phenotypes. In GSEA, genes are sorted by correlation, and an ordered list is generated. Each gene in the gene set is checked for enrichment in either the upper or lower portion of the list to assess the impact of coordinated gene changes on phenotypic changes. GSEA was performed for all DEGs, with genes from C2.all.v7.2.symbols.gmt and the HPO as reference gene sets. Gene Set Variation Analysis (GSVA) is a parameter-free, unsupervised method that calculates the enrichment score of a specific gene set without predefined sample grouping. GSVA quantifies gene enrichment results in the transformed expression matrix, reflecting the characteristics of the gene set. Using C2.all.v7.2.symbols.gmt as the reference gene set, GSVA was performed on the batch-corrected expression profiles to generate a quantified GSVA score for each gene set. Differential analysis was conducted using the ‘limma’ R package based on the grouping information of DKD patients and normal controls. Gene sets were considered significantly enriched if the *p* value was <0.05 and the false discovery rate (FDR) was <25%.

### Functional and pathway enrichment analysis

To explore the biological processes involved in the identified DEGs, gene enrichment analysis was conducted. A Venn diagram was drawn based on the intersection between the identified DEGs and the Immport database. The ‘ClusterProfiler’ R package was used to identify the biological processes and protein interaction network information for the top 10 enriched DEGs in Disease Ontology (DO) [28], Gene Ontology (GO) [29], and Kyoto Encyclopedia of Genes and Genes Pathway (KEGG) [30] enrichment analysis. These functional and pathway analyses are widely used to identify significantly annotated/enriched/overrepresented functions, classes, terms, and pathways associated with the DEGs. The ‘GOSemSim’ R package was used for semantic analysis to demonstrate the significance of immune-related hub DEGs in GO functional analysis, including annotations of biological processes (BP), cell components (CC), and molecular functions (MF). The results were visualized using a Raincloud diagram generated with the ‘ggplot2’ R package. Statistical significance was set at a *p* value < 0.05.

### Screening for candidate gene biomarkers

To assess the prognostic value of potential immune-related genes in DKD, 13 identified DEGs were further screened using LASSO regression analysis and the support vector machine recursive feature elimination (SVM-RFE) algorithm, followed by verification using the receiver operating characteristic (ROC) curve. The batch-corrected GSE1009 and GSE96804 datasets were used as the training set, and the batch-corrected GSE30528 dataset was used as the validation set. First, the least absolute shrinkage and selection operator (LASSO) algorithm was applied to reduce the number of DKD biomarkers in the training set. The LASSO penalty regression model, using the least classification error λ, was employed to determine the variables. The SVM-RFE algorithm was also applied. The SVM-RFE algorithm constructs feature ranking coefficients based on the weight vector generated by the SVM during training. The feature with the smallest ranking coefficient is removed in each iteration, and the final ranking of all features is obtained. Finally, to further evaluate the predictive accuracy of the identified candidate gene biomarkers, the ROC curve was used, and area under the curve (AUC) for each candidate gene was analyzed to predict DKD status in the GSE30528 validation cohort.

### CIBERSORT immune infiltration analysis

The proportion of immune infiltration, including 22 types of immune cells, was analyzed using the CIBERSORT [15] algorithm in the three datasets. The correlation between different immune cells was also calculated. The fraction of immune-infiltrating cell subtypes was compared between DKD patients and normal subjects. Gene biomarkers were then identified, and the correlation between these selected genes and immune infiltration levels was analyzed. The percentage of each immune cell type in the three datasets was calculated. The Pearson correlation coefficient and the significance level (*p* value) were computed, with *p* < 0.05 considered significant. Finally, a lollipop plot illustrating the correlation between each candidate gene marker and immune cells was generated using the ggplot2 R package.

### RT-PCR validation of candidate gene biomarkers

To validate the findings from the bioinformatics analysis, kidney tissue from five 20-week-old male db/db mice and five db/m mice (C57BLKS/J db/db) was collected for RT-PCR confirmation. All mice were purchased from the Experimental Animal Center of Nanjing Medical University. The mice were housed at the Experimental Animal Center of Anhui Medical University under optimal conditions (room temperature of 24 ± 1 °C, humidity 60%, alternating 12-h light and dark cycle). All experiments followed the ARRIVE guidelines (https://arriveguidelines.org). Mice were sacrificed according to the NIH Guide for the Care and Use of Laboratory Animals. The study protocol was approved by the Ethics Committee of Anhui Medical University (approval number: LLSC20211308). Total RNA was isolated from the whole renal cortex tissue using the Magen HiPure Total RNA Mini Kit (Magen, Guangzhou, China). RNA samples were reverse-transcribed to cDNA, and RT-PCR was performed using MonScript^™^ RTIII All-in-One Mix (with dsDNase) (Monad Biotech Co., Ltd., Shanghai, China). All primer sequences are listed in Supplement Table S1. GAPDH was used as an internal reference. Relative mRNA expression was calculated using the 2-ΔΔ Ct. All tests were performed in triplicate. The Wilcoxon Rank Sum Test was used to compare expression values, with *p* < 0.05 considered statistically significant.

### PPI network creation and immune-related hub gene identification

The protein-protein interaction (PPI) network of DEGs was constructed using the Search Tool for the Retrieval of Interacting Genes (STRING) [31] online database (http://string‐db.org; version 10.5; accessed on 01 February 2022). Interactions between DEGs with a combined score >0.7 were considered significant. Cytoscape (version 3.6.1) [32], an open-source bioinformatics software, was used to improve the quality of the PPI network and visualize molecular interaction networks. The Cytoscape plugin CytoHubba was employed to construct the PPI network using the maximum correlation criterion (MCC) algorithm and to identify hub genes. The PPI network consists of nodes (representing proteins) and edges (representing interactions). A node with a higher number of significant interactions or edges is considered a top-ranked hub gene. The top ten ranked genes were selected for the hub gene set. The expression differences of these hub genes across immunotypes were analyzed, and box plots were generated using the ggplot2 R package.

### Interaction networks for key immune-related hub genes

The overlap genes between the hub gene set and candidate gene biomarkers were selected as key immune-related DEGs. The interaction network of these key genes was analyzed from three aspects: target gene-transcription factor relationships, experimentally validated microRNA (miRNA)-mRNA interactions, and the relationship between key immune-related genes and drugs. Target gene-transcription factor enrichment analysis was performed using the ChEA3 online tool [33] (https://maayanlab.cloud/chea3/). Target gene – miRNA enrichment analysis was based on the miRTarget [34] database (http://multimir.ucdenver.edu/) and TarBase [35] (https://dianalab.e-ce.uth.gr/html/diana/web/index.php?r=tarbasev8/index). Target gene-drug composition enrichment analysis was conducted using the DGIDB [36] database (https://dgidb.genome.wustl.edu/). The collected interaction data were imported into Cytoscape software for network visualization.

## Results

### Quality control and data standardization

**Figure 2.**
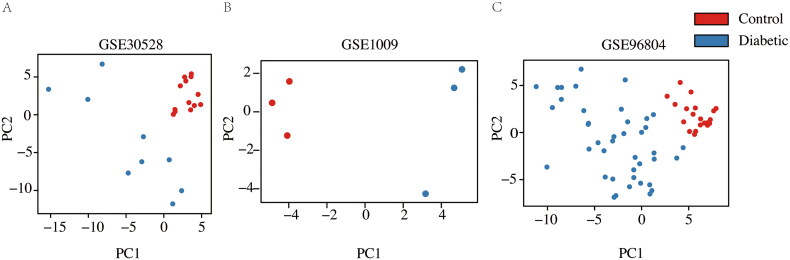
Principal component analyses (PCA) of gene expression between the DKD and normal control groups across three datasets. (A) PCA in GSE30528. (B) PCA in GSEB1009. (C) PCA in GSE96804. Blue indicates the DKD group, and red indicates the normal group.

**Figure 3.**
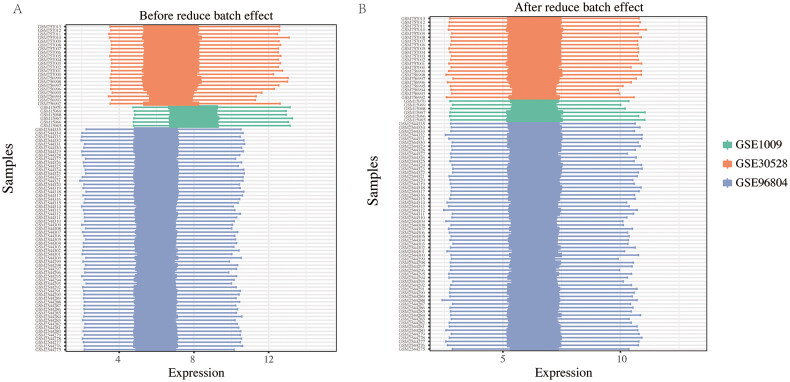
Box plots of the three datasets before and after batch correction. (A) Box plot of the three datasets before batch correction. (B) Box plot of the three datasets after batch correction. Green bar charts represent GSE1009, red bar charts represent GSE30528, and blue bar charts represent GSE96804.

To explore DEGs between DKD patients and normal individuals in each dataset, the raw data (GSE1009, GSE30528, and GSE96804) was standardized by log2 transformation of the expression values. Sample information was organized into a table, and the grouping information was recorded. A total of 89 samples (DKD = 53, normal = 36) were included in the datasets. The quality of the microarray data was assessed using principal component analysis (PCA) in each dataset. The results showed significant differences in gene expression between DKD patients and normal subjects (Figure 2). After merging the three datasets, 7401 overlapping genes were identified. Quality assessment of the overlapping gene expression profile, before and after batch correction, indicated that the samples clustered together after removing the batch effect (Figure 3). In this study, to ensure data consistency, we conducted PCA on individual datasets and employed ComBat for batch correction. We acknowledge that merged PCA visualization would further enhance interpretability. Future studies will prioritize this approach to complement our analysis.

### Identification of immune-related DEGs in DKD

**Figure 4.**
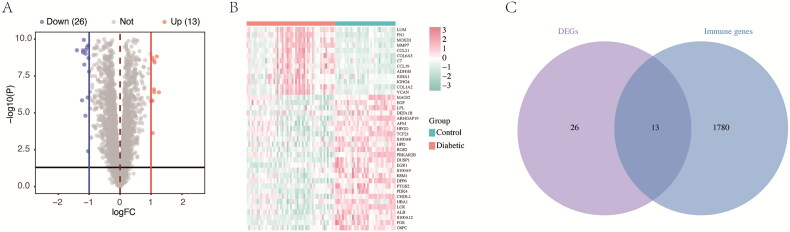
Diagrams of DEGs in the batch-corrected datasets. (A) Volcano plots show the distributions of all DEGs, with 13 upregulated genes (red dots) and 26 downregulated genes (blue dots). Genes with no significant expression differences are marked as grey dots. (B) Heatmap of DEGs screened using the limma ‘R’ package. Red areas represent highly expressed genes, and green areas represent lowly expressed genes in glomeruli from DKD patients compared with normal controls. (C) Venn diagram showing the overlap of DEGs and immune-related genes from the Immport database.

Using the limma ‘R’ package on batch-corrected data, 39 DEGs were identified based on adjusted *p* < 0.05 and log2|FC| > 1 (Figure 4(A,B)). Among them, 13 were associated with immune function in DKD by comparison with the Immport Database (Figure 4(C)).

### Immune subtype analysis

**Figure 5.**
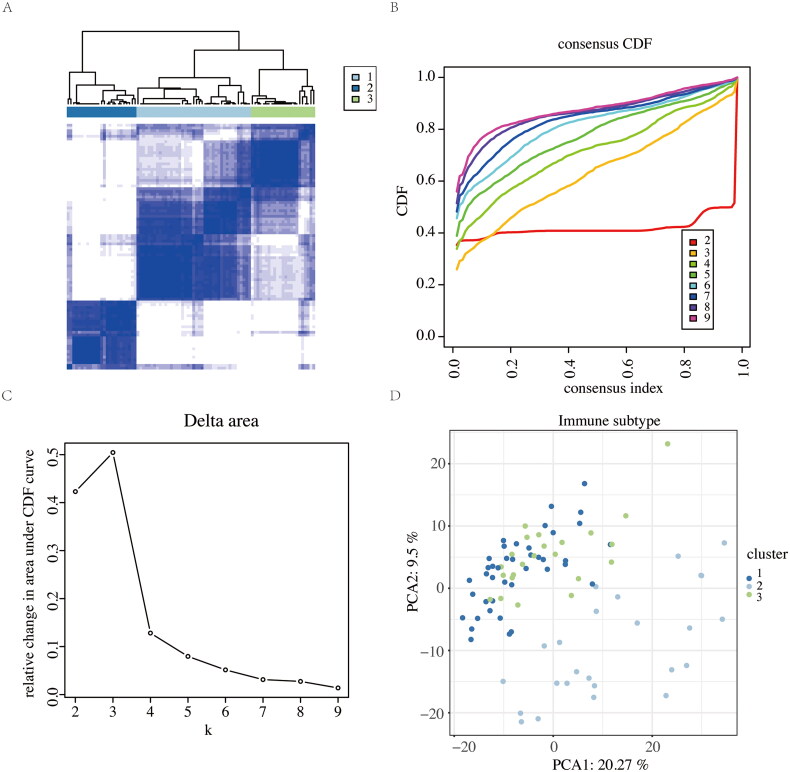
Consistent clustering analyses of the 13 identified immune-related DEGs. (A) Heatmap using consistent clustering analyses with *K* = 3. (B) Consistent cumulative distribution function (CDF) plot. (C) Delta Area plot. (D) PCA plot.

To explore the underlying biological characteristics of distinct immunophenotypes, differential consistent clustering analysis was performed on the 13 identified immune-related DEGs. Heatmaps (Figure 5(A)), Cumulative Distribution Function (CDF) maps (Figure 5(B)), and Delta Area maps (Figure 5(C)) were used to determine the optimal cluster number (*K* = 3). Principal component analysis (PCA) based on these clusters revealed three distinct immune subtypes (Figure 5(D)). The three immune subtypes were: Cluster 3 (High Fibro-Inflammatory Subtype): Characterized by pronounced upregulation of extracellular matrix (ECM)-related hub genes (e.g., FN1, COL1A1, LOX), and reflects active fibrotic progression and inflammatory infiltration. Patients in this cluster likely exhibit accelerated DKD progression driven by ECM remodeling and macrophage-T cell crosstalk; Cluster 2 (Low Fibro-Inflammatory Subtype): Marked by subdued expression of fibrotic and inflammatory mediators. This subtype suggests a quiescent immune microenvironment, potentially corresponding to early-stage or stabilized DKD; Cluster 1 (Intermediate Subtype): Displays transitional gene expression profiles between Clusters 2 and 3, indicating dynamic shifts in immune-fibrotic balance.

### EPIC immune infiltration analysis

**Figure 6.**
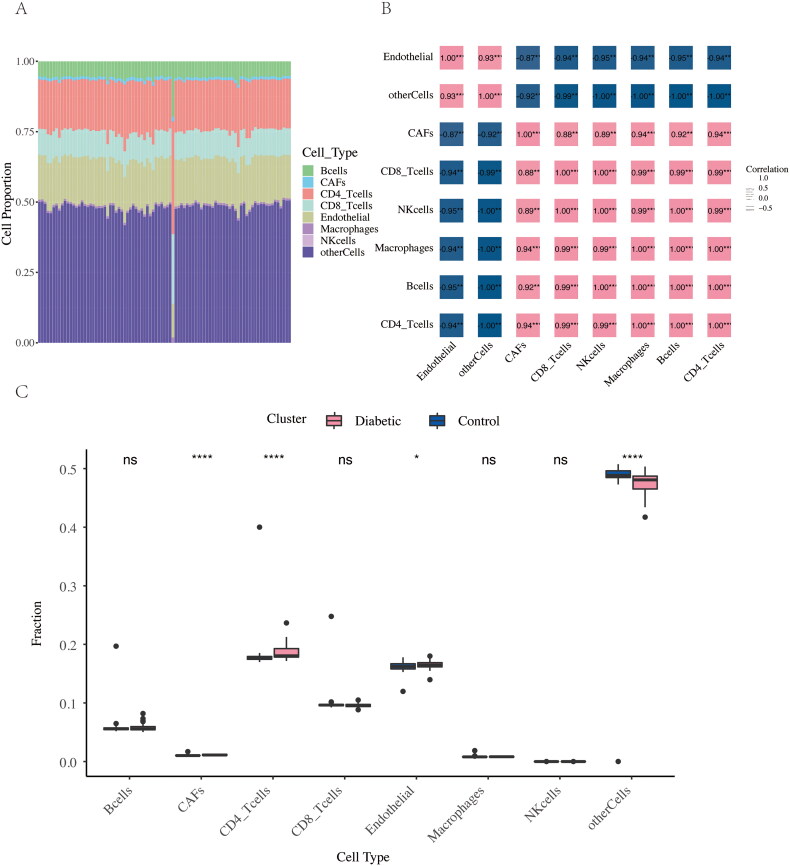
Immune cell infiltration analysis using EPIC. (A) Stacked bar graphs showing the immune cell composition based on EPIC. (B) Correlation between different infiltrated immune cells. (C) Box plot showing the distribution of infiltrated immune cells between DKD and normal subjects (*p value < 0.05; ****p value < 0.0001).

To evaluate immune cell infiltration, the EPIC database (https://gfellerlab.shinyapps.io/EPIC_1-1/) was applied to the merged expression profiles. First, we analyzed the immune cell composition in each gene set, as shown in the stacked bar graphs (Figure 6(A)). Next, correlations between different infiltrating immune cells in the dataset were explored (Figure 6(B)). Finally, a differential analysis of immune cell expression between DKD samples and normal controls was conducted, as shown in the box plot (Figure 6(C)). The correlation heatmap revealed that endothelial cells were strongly correlated with other cell types. T cells were strongly correlated with cancer-associated fibroblasts (CAFs), natural killer (NK) cells, and macrophages. The infiltration levels of CAFs and CD4+ T cells were significantly different between DKD patients and normal controls.

### Gene and GSEA

**Figure 7.**
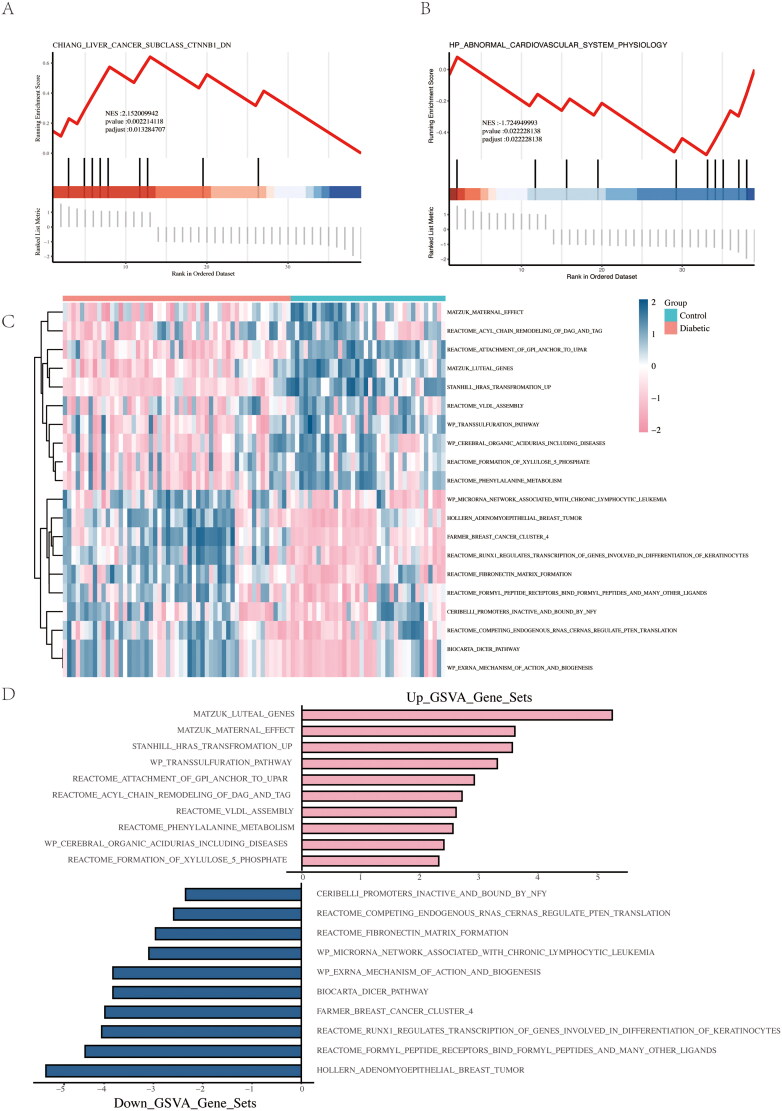
Results of GSEA and GSVA. (A) DEGs enriched in the C2 gene set by GSEA. (B) DEGs enriched in the HPO gene set by GSEA. (C) Heatmap of DEGs identified by GSVA in the batch-corrected profiles. (D) Histogram of upregulated and downregulated DEGs identified by GSVA in the batch-corrected profiles.

We selected C2.all.v7.2.symbols.gmt and HPO as reference gene datasets for GSEA, using the DEGs from our differential analysis. We found that the differentially expressed gene set was linked to gene set pathways related to liver cancer and abnormal cardiovascular diseases (Figure 7(A,B), Supplement Table S2). After batch-correcting the expression profiles, we performed GSVA to quantitatively obtain the GSVA scores for each gene set. Then, based on the grouping of DKD patients and normal individuals, we used limma for differential analysis. We defined the top 10 gene sets with a *p* value < 0.05 and log2|FC| as the differentially expressed gene sets. Subsequently, we plotted a heatmap of gene-set differences (Figure 7(C)) and a bar chart of up- and down-regulated genes (Figure 7(D), Supplement Table S3). We discovered that the differentially expressed gene set was associated with gene sets like the DICER pathway, phenylalanine metabolism, and breast cancer development. This implies that these genes could be crucial in disease onset and progression, and might significantly impact diseases and even cancer.

### Functional and pathway enrichment analysis

Using the clusterProfiler in R, gene enrichment analysis was carried out on DO, GO, and KEGG databases for the identified DEGs. The top 10 significantly enriched DEGs were presented in various plots (Supplement Figure S1 and Table S4). While the enrichment analysis was based on a small subset of immune-related DEGs (*n* = 13), which may limit the statistical robustness of pathway associations, such exploratory analyses are widely used in biomarker discovery studies to provide preliminary insights into disease-related pathways [37,38]. GO analysis showed that immune-related DEGs were mainly involved in leukocyte and neutrophil chemotaxis, neutrophil migration, defense against fungi and bacteria, etc. The key cellular components included neurosecretory granules, and molecular functions covered RAGE receptor binding. KEGG enrichment indicated that the DEGs were concentrated in cytokine-cytokine receptor interactions and cancer-related pathways. DO analysis associated these DEGs with kidney and urinary system diseases. Friends analysis, *via* GOSemSim R package, showed in the Raincloud diagram (Supplement Figure S1) that FN1 was the most influential gene, while MMP7 had the least impact among the immune-related DEGs. FOS and ALB were also highly significant.

### Screening for candidate gene biomarkers

**Figure 8.**
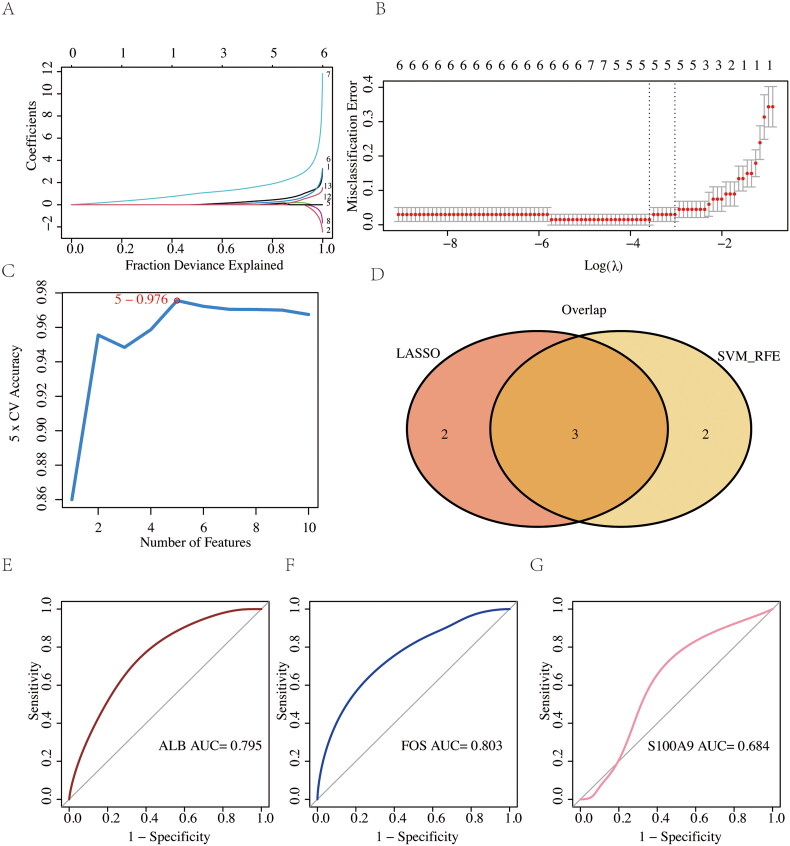
Potential gene biomarker screening for DKD based on the LASSO penalty regression model and SVM-REF algorithm, validated by ROC curve. (A) The LASSO penalty regression model in the training set. (B) The optimal λ in the training set. (C) SVM-REF algorithm in the training set. (D) Overlapping DEGs associated with DKD in the LASSO regression model and SVM-REF (including ALB, FOS, and S100A9). (E-F) ROC curve for ALB, FOS, and S100A9 in the validation set (AUC = 0.795, 0.803, 0.684, respectively).

To identify potential immune-related prognostic genes in DKD, 13 identified DEGs were further screened using LASSO regression analysis and the SVM-REF algorithm, followed by validation using the receiver operating characteristic (ROC) curve. The GSE1009 and GSE96804 datasets, batch-corrected, were used as the training set, while GSE30528 was used as the validation set. In the LASSO penalty regression model (Figure 8(A)), the least classification error λ was used to select the variables, resulting in 5 candidate genes (Figure 8(B)). The SVM-REF algorithm identified 5 significant DEGs (Figure 8(C)). Finally, based on both the LASSO and SVM-RFE algorithms, 3 overlapping genes were identified as potential biomarkers associated with DKD: ALB, FOS, and S100A9 (Figure 8(D)). ROC curves showed that the AUC for ALB, FOS, and S100A9 in predicting DKD was 0.795, 0.803, and 0.684, respectively, suggesting their potential as diagnostic biomarkers for DKD (Figure 8(E,F)).

### CIBERSORT immune infiltration analysis

**Figure 9.**
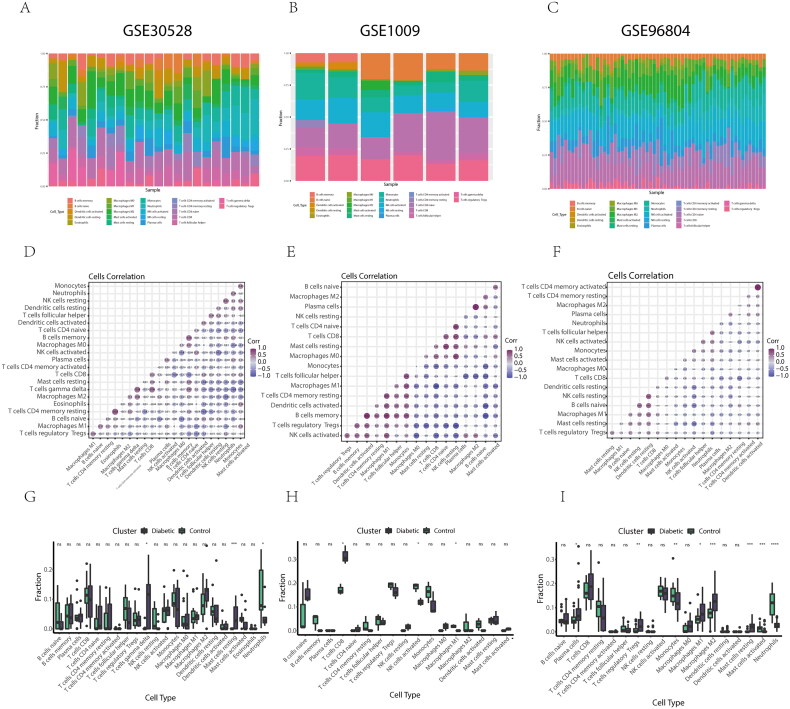
Subtypes of immune cell infiltration in the three datasets analyzed by the CIBERSORT algorithm. (A-C) Stacked bar graphs of immune cell infiltration in GSE30528, GSE1009, and GSE96804, respectively. (D-F) Correlation between each infiltrated immune cell in GSE30528, GSE1009, and GSE96804, respectively. (G-I) Box plots showing immune infiltration levels between DKD patients and normal subjects in GSE30528, GSE1009, and GSE96804, respectively (green box represents normal subjects, black box represents DKD patients).

**Figure 10.**
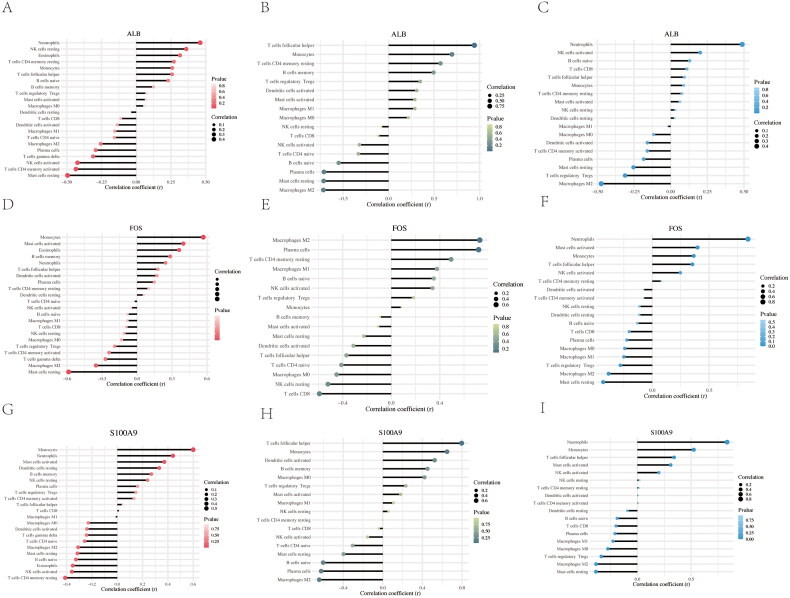
Correlation between ALB, FOS, and S100A9 with different infiltrated immune cells across the three datasets. (A-C) The correlation between ALB and infiltrated immune cells in GSE30528, GSE1009, and GSE96804, respectively. (D-F) The correlation between FOS and infiltrated immune cells in GSE30528, GSE1009, and GSE96804, respectively. (G-I) The correlation between S100A9 and infiltrated immune cells in GSE30528, GSE1009, and GSE96804, respectively.

To further explore immune infiltration across the three datasets, the proportion of 22 immune cell subtypes was analyzed using the CIBERSORT algorithm, with results visualized in Figure 9(A–C). The correlations between different infiltrating immune cell subtypes are shown in Figure 9(D–F). The differences in immune cell infiltration between DKD patients and normal controls in the three datasets are summarized in Figure 9(G–I). Lollipop maps illustrating the correlation between each potential gene biomarker (ALB, FOS, and S100A9) and immune cells are presented in Figure 10(A–I). These maps revealed strong correlations between B cells and CD4 T cells, as well as CD4+ T cells and natural killer (NK) cells. The levels of CD8+ T cells were significantly different between DKD patients and normal subjects. ALB, FOS, and S100A9 showed strong correlations with macrophages, T cells, and NK cells, indicating their involvement in the dynamic regulation of immune homeostasis.

### RT-PCR validation for candidate gene biomarkers

**Figure 11.**
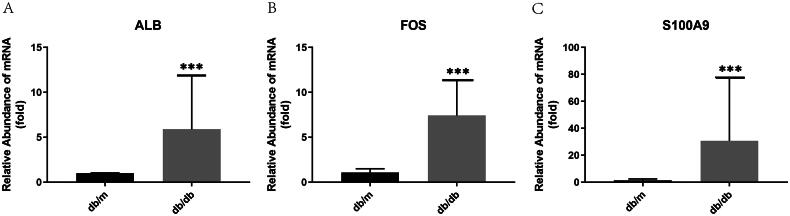
RT-PCR validation of the expression of the three candidate gene biomarkers in kidney tissue from 5 db/db mice and 5 db/m mice. Results were presented as mean ± SD. (***p value < 0.001).

The RT-PCR validation results indicated relatively high expression levels of the three candidate gene biomarkers (ALB, FOS, and S100A9) in kidney tissue from type 2 db/db mice compared with db/m mice (all *p* < 0.001) (Figure 11(A–C)). These findings were consistent with the results of the bioinformatics analysis of microarray hybridization. Raw data and analysis scripts are available in supplement files.

### PPI network creation and immune-related hub genes identification

**Figure 12.**
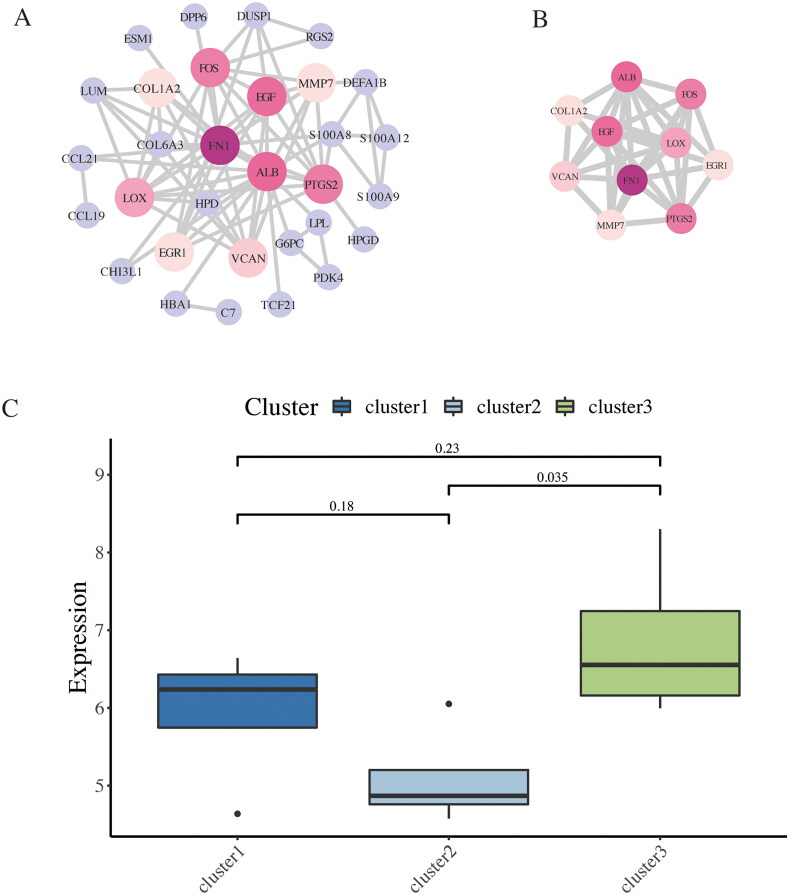
Identification of hub genes based on PPI network and immune cluster analysis. (A) The PPI network with 31 nodes was constructed based on the STRING database. (B) The network of the top 10 ranked hub genes. (C) Comparison of the expression level of hub genes in different immune clusters.

A PPI network of DEGs was constructed using the Search Tool for the Retrieval of Interacting Genes (STRING) database (https://www.string-db.org). The PPI network, comprising 31 nodes, is shown in Figure 12(A). The top 10 hub genes, selected by the MCC method from the cytoHubba plugin in Cytoscape, include FN1, EGF, PTGS2, ALB, FOS, LOX, EGR1, COL1A2, VCAN, and MMP7 (Figure 12(B)). These genes may play key roles in the immune response during DKD progression. The expression levels of these hub genes in the three immune clusters were compared using the ggplot2 R package, and the results are presented as a box plot in Figure 12(C). A significant difference in expression was observed between cluster 2 and cluster 3. These two clusters appear to be the most important immune clusters in DKD patients.

### Interaction networks for key immune-related hub genes

**Figure 13.**
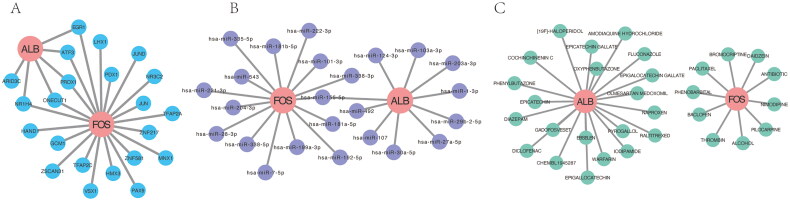
Transcription factor, miRNA, and drug composition interaction network of ALB and FOS. (A) Target gene-transcription factor interaction network of ALB and FOS. (B) Target gene-miRNA interaction network of ALB and FOS. (C) Target gene-drug composition interaction network of ALB and FOS.

Among the 10 hub genes and three potential gene biomarkers, ALB and FOS were identified as overlapping genes and key immune-related hub genes in DKD. Therefore, we further analyzed the interaction networks involving the target gene-transcription factor, target gene-miRNA, and target gene-drug composition for ALB and FOS. These interaction relationships were visualized using Cytoscape and are displayed in Figure 13.

## Discussion

Despite decades of clinical efforts targeting glycemic control and renin-angiotensin system blockade, diabetic kidney disease (DKD) remains responsible for nearly half of global ESKD cases [39], underscoring critical gaps in current therapeutic strategies. Accumulating evidence now implicates immune dysregulation and chronic inflammation as central mediators of DKD progression – a paradigm shift that necessitates systematic exploration [2,6,9,40]. Our multi-omics integration of glomerular transcriptomes (*n* = 89 samples) identified 13 immune-related DEGs, with pathway analysis revealing predominant involvement in cytokine-cytokine receptor interactions and leukocyte chemotaxis. Combining machine learning prioritization with experimental validation, ALB and FOS were identified as core regulators of macrophage-T cell interplay (AUC: 0.803 and 0.795, respectively; *p* < 0.05 in db/db mice). These findings establish a mechanistic link between genomic alterations and inflammatory microenvironment remodeling, proposing actionable targets for immunomodulatory interventions in DKD management.

In this study, we used a novel analytical framework with the CIBERSORT algorithm to analyze immune cell infiltration dynamics. Unlike prior research focusing on isolated immune cell populations or specific pathways, our approach systematically quantified immune cell subtype proportions. Although both EPIC and CIBERSORT are popular immune cell analysis tools, their applications vary depending on the context. EPIC, optimized for tumor microenvironments, has limited validation in non-neoplastic tissues like the kidney, while CIBERSORT uses curated immune cell signatures to accurately estimate immune cell abundances in heterogeneous samples, enhancing reproducibility in renal studies. By integrating three datasets from GEO, we identified significant differences in immune cell subtypes between DKD patients and healthy controls. We employed both CIBERSORT and EPIC, combining their respective advantages in different cell population analyses to comprehensively depict the immune infiltration network in DKD patients. CIBERSORT, which focused on refining the analysis of lymphocyte subsets, enabled us to understand the proportional changes and roles of different lymphocytes in the disease. Through this analysis, we found genes such as ALB and S100A9, closely related to disease progression. These genes might promote disease progression by regulating macrophages and NK cells. S100A9, especially associated with the inflammatory process, could affect the activity and distribution of immunomodulatory cells. Meanwhile, EPIC excelled in analyzing stromal cells such as CAFs and endothelial cells, allowing us to observe their abnormal behaviors in immune infiltration. Using EPIC, we discovered abnormal infiltration patterns of CD4+ T cells and CAFs, indicating a chronic inflammation-dominated microenvironment. This suggests CD4+ T cells may interact with CAFs to continuously activate and maintain inflammatory signals, influencing DKD’s immune characteristics and potentially related complications. The combined use of CIBERSORT and EPIC has provided a comprehensive view of the immune infiltration network in DKD, highlighting the importance of both lymphocyte subsets and stromal cells in the disease process. By integrating the results of CIBERSORT and EPIC, we constructed a detailed immune environment picture, showing how multiple cell types interact in DKD to impact the disease process. This provides a new perspective for understanding DKD’ pathological mechanism and a basis for identifying potential therapeutic targets. For example, EPIC analysis showed distinct patterns between CAFs and CD4+ T cells, suggesting gene-mediated immune infiltration regulation, which was supported by CIBERSORT’s gene-immune cell correlations. Our approach not only clarified the immune microenvironment’s role in DKD pathogenesis but also emphasized the functional interdependencies among immune cell populations, especially characterizing the interaction between CD4+ T cells and NK cells, uncovering synergistic mechanisms that may trigger inflammatory cascades in DKD.

Contrary to the traditional view of DKD as a ‘bland’ glomerulopathy, our data implicate immune responsiveness and inflammatory cascades as pivotal contributors to its pathogenesis. Numerous studies have confirmed that immune cell infiltration is a prominent feature of DKD [9,12]. Previous evidence suggests that immune responses involve both innate and adaptive immune components in the development of DKD. The innate immune system comprises cellular and humoral mediators that defend against pathogens and internal danger signals, and also regulate the activation of the adaptive immune response [41]. A recent single-cell RNA sequencing study on cryopreserved human diabetic kidney samples revealed that, compared to controls, there was a significant increase in infiltrating immune cells, with leukocyte levels approximated to be 7 to 8 times higher, consistent with our findings and previous studies. Leukocytes are a plausible non-kidney source of KRIS proteins, which consist of molecules primarily involved in the etiology of renal function loss and innate immune responses, many of which are expressed by monocytes [11]. Our analysis showed that T cells and macrophages prominently infiltrated the kidneys in DKD. These findings highlight potential therapeutic targets for immune modulation, such as disrupting T cell recruitment *via* ICAM-1/LFA-1 blockade – a strategy already under investigation in inflammatory diseases. In DKD rat models, early infiltration of helper T cells (CD4+ T cells) was observed, followed by a later influx of macrophages and cytotoxic T cells (CD8+ T cells) [2,42]. The migration of T cells into the kidney is likely facilitated by interactions between the constitutive T cell lymphocyte function-associated antigen-1(LFA-1) and intercellular adhesion molecule-1 (ICAM-1), which are expressed on renal epithelial, endothelial, and mesangial cells [2,43]. Similarly, in ICAM-1 deficient db/db mice, the migration of T cells into the kidney was significantly reduced [44].

KEGG pathway analysis revealed that the 13 identified DEGs were predominantly enriched in cytokine-cytokine receptor interactions and oncogenic pathways, both central to inflammatory pathophysiology. Cytokines, as polypeptide mediators, exert pleiotropic effects across diverse cell types [45]. These molecules mediate intercellular communication *via* autocrine, paracrine, and juxtacrine mechanisms, critically regulating immune cell functionality and systemic inflammatory responses. Previous research has confirmed the role of inflammation and immune activation in the pathogenesis of DKD by implicating chemokines, such as monocyte chemoattractant protein-1 (MCP-1) and inflammatory cytokines, including tumor necrosis factor-alpha (TNF-α) [40,46]. For instance, specific blockade of the MCP-1 receptor, C-C motif chemokine receptor 2 (CCR2), reduced albuminuria in diabetic mice with a human CCR2 transgene, while specific TNF-α inhibitors decreased proteinuria in streptozotocin (STZ)-induced rats. Other cytokines, such as interleukin-1, −6, and −18 (IL-1, IL-6, IL-18), ICAM-1, vascular cell adhesion protein 1 (VCAM-1), adiponectin, peroxisome proliferator-activated receptors (PPAR-α, PPAR-γ, PPAR-δ), E-selectin, leptin, endothelial cell-selective adhesion molecule (ESAM), and matrix metalloproteinase-2 (MMP-2), are considered primary regulators of inflammation in DKD. Substantial evidence suggests these factors strongly correlate with the onset and progression of renal injury in diabetic patients [47]. Consistent with previous studies, renal cells, such as those in the glomeruli, secrete pro-inflammatory cytokines that activate a wide variety of cytokine-cytokine receptor interaction signaling and molecular pathways, leading to inflammatory responses that contribute to the progression of renal damage.

Chair et al. [48] investigated genetic variants across six major immune response-related pathways through systematic meta-analysis of association studies from PubMed and HuGE Navigator, identifying specific gene variants linked to DKD risk. Notably, our findings diverge from Chair et al.’s study [48], potentially attributable to methodological disparities: while Chair et al. [48] focused on germline genetic associations, our approach prioritized transcriptomic alterations and immune microenvironment dynamics, reflecting distinct yet complementary biological perspectives. Recent advances in bioinformatics approaches, such as those exemplified by Zhang et al. [49] and Fu et al. [50], have further highlighted the heterogeneity of immune mechanisms in DKD. For instance, Zhang et al. [49] identified SLIT3, PDE1A, and CFH as immune-associated hub genes through integrated scRNA-seq and transcriptomic analyses, corroborating our emphasis on immune cell infiltration and pathway dysregulation. Similarly, Fu et al. [50] demonstrated dynamic macrophage polarization states during early DKD progression, aligning with our observations of immune cell heterogeneity and its correlation with disease severity. In terms of research methodologies, we obtained gene expression profiles from the GEO database and employed a variety of analytical techniques to identify key genes based on gene expression levels and immune cell infiltration. In contrast, Chair et al.’s study [48] focuses on genetic associations related to specific gene variations documented in existing databases. The available data in their study is more limited and specialized, primarily concerning association studies linking specific gene variations to DKD. These differences in data sources and analytical methods may explain the discrepancies in gene associations with DKD. From a biological perspective, the key genes identified in our study, such as ALB and FOS, along with other immune-related hub genes, may operate through different mechanisms in DKD compared to those highlighted in Chair et al.’s study. Notably, while Zhang et al. [49] validated the diagnostic potential of immune-associated genes through longitudinal cohort analyses, our findings extend this paradigm by elucidating the functional interplay between ALB/FOS and specific immune pathways. Our research suggests that ALB and FOS may be involved in immune cell regulation and kidney tissue repair *via* specific signaling pathways, whereas the genes examined in Chair et al.’s study [48] may influence DKD progression through alternative immune mechanisms. Furthermore, the observed differences in immune cell infiltration between DKD patients and healthy controls may provide a biological explanation for the divergent results. In summary, although our findings differ from Chair et al.’s study, these discrepancies offer new insights and potential avenues for exploring the immune mechanisms involved in DKD. The complementary evidence from Zhang et al. [49] and Fu et al. [50] underscores the necessity of integrating multi-omics approaches to unravel the spatiotemporal complexity of immune dysregulation in DKD. Further research will be crucial for identifying key genes and immune pathways linked to this condition, ultimately offering new targets for diagnosis and therapeutic intervention.

To further validate our findings, an RT-PCR analysis on the three potential gene biomarkers was performed. The results showed that all three genes were upregulated in the kidney tissue of type 2 diabetic db/db mice compared to db/m mice, a well-established model for human DKD with a long history of use in research [51]. The genes ALB, FOS, and S100A9 were identified as potential biomarkers, demonstrating significant diagnostic value for DKD. Serum albumin (ALB), synthesized in the liver, encodes the most abundant protein in vertebrate plasma. Albumin is typically present in the blood and filtered by the kidneys. When the kidneys are impaired, abnormal amounts of albumin may leak into the urine. Consequently, the concentration of albumin in serum synthesis is reduced during inflammation or inadequate protein intake. Additionally, numerous studies have shown that levels of various chemokines and pro-inflammatory cytokines, such as MCP-1, TNF-α, and IL-18, are elevated in the renal tissue of diabetic patients, correlating with the progression of urinary albumin excretion [41,52]. Beyond its classical role as a marker of hypoalbuminemia, ALB has recently emerged as an active mediator of immune dysregulation. Zhang et al. [49] demonstrated that tubular ALB overexpression exacerbates chemokine-driven immune infiltration, aligning with our findings of its correlation with T cell and macrophage recruitment. These observations reposition ALB from a passive biomarker to a multifaceted player in DKD pathogenesis. Low serum ALB is a common feature in patients with ESKD and has predictive value in DKD [53,54].

The transcription factor activator protein-1 (AP-1) is a potent immune system regulator and a heterodimer composed of c-Fos and c-Jun. AP-1 is expressed in various mucosal cell types, including fibroblasts, endothelial cells, and macrophages [55,56]. While AP-1 generally acts as an activator of pro-inflammatory genes, the role of c-Fos appears to be the opposite [57]. In macrophages, c-Fos has been shown to suppress the expression of inducible nitric oxide synthase (iNOS) and pro-inflammatory cytokines, such as TNF, IL-6, and IL-12, while simultaneously increasing the expression of anti-inflammatory genes, including IL-10, suppressor of cytokine signaling 1 (SOCS1), and SOCS3 ^55^. Additionally, c-Fos plays a significant role in the polarization of THP-1 monocytes into macrophages [58]. It may regulate macrophage subtype polarization by inhibiting the formation of M1 macrophages and promoting the transition from M1 to M2 macrophage polarization [58]. Wei et al. [59] conducted a study on hub genes in early DKD based on weighted gene co-expression network analysis, which showed that FOS was downregulated in early DKD. The discrepancies in FOS expression observed between our study and existing literature may be attributed to differences in disease stages, methodological variations, and the biological complexity of the condition. To clarify the role of FOS in DKD, further research with larger sample sizes and diverse methodologies is required. A more comprehensive examination of stage-specific mechanisms and their clinical implications could enhance our understanding and inform therapeutic strategies.

S100A9 is a calcium- and zinc-binding protein involved in regulating inflammatory and immune responses [60]. It stimulates neutrophil chemotaxis and adhesion, enhancing phagocytic activity and bactericidal function by activating the PI3K/AKT, SYK, and ERK1/2 pathways. It also promotes neutrophil activation through a MAPK-dependent mechanism [60,61]. Intracellularly, S100A9 aids the transport and metabolism of arachidonic acid in leukocytes, regulates the cytoskeleton during phagocyte migration, and activates neutrophil NADPH oxidase [62]. S100A9 activates NADPH oxidase by enhancing the enzyme complex at the cell membrane and transferring arachidonic acid, an essential cofactor, to the enzyme complex. Its role in neutrophil activation and NADPH oxidase assembly suggests pharmacological inhibition could attenuate oxidative stress and inflammation in diabetic kidneys a strategy paralleling recent successes in targeting DAMPs in autoimmune diseases. Furthermore, S100A8 facilitates enzyme assembly by directly binding to NCF2/P67PHOX [63,64]. Extracellularly, S100A9 exhibits pro-inflammatory, antibacterial, oxidant-scavenging, and apoptosis-inducing functions [65]. Its pro-inflammatory roles include leukocyte recruitment, promotion of cytokine and chemokine production, and regulation of leukocyte adhesion and migration [60,66]. S100A9 also functions as a danger-associated molecular pattern (DAMP) molecule, stimulating innate immune cells by binding to pattern recognition receptors such as TLR4 and AGER. In our bioinformatics analysis, S100A9 demonstrated strong predictive value for DKD. A study focusing on DKD identified S100A9 as a potential biomarker in urinary extracellular vesicles for early-stage DKD, further supporting its utility in developing point-of-care diagnostic devices for early detection [67]. ALB, FOS, and S100A9 play pivotal roles in DKD. However, larger sample sizes and clinical studies are needed to further investigate their mechanisms.

The current study has several notable limitations. Firstly, to comprehensively elucidate immune infiltration in DKD, data from DKD patients with varying disease durations are required. Secondly, while we validated the expression levels of the three identified potential diagnostic biomarkers in kidney samples from db/db mice and db/m mice, the RT-PCR validation was performed on whole renal cortex tissue rather than isolated glomeruli. Although this approach captures broader expression trends relevant to DKD, as cortical tissue includes glomeruli, tubules, and interstitial compartments, it may dilute glomerulus-specific signals. Future studies should employ laser-capture microdissection (LCM) of glomeruli for targeted validation to address this limitation. Further validation is needed to confirm their localization and expression in immune cells. Moreover, our research has focused on the overall expression of ALB, FOS, and S100A9 in db/db mice, without delving into their expression patterns in specific renal cell types. Analyzing these patterns using publicly available single-cell datasets (such as https://humphreyslab.com/SingleCell/) could provide deeper insights. In addition, the relationship between the expression of ALB, FOS, and S100A9 and key renal function indicators in db/db mice, including serum creatinine, urinary albumin, urinary albumin-to-creatinine ratio, and estimated glomerular filtration rate (eGFR), has not been explored in detail in this study. Understanding these associations is crucial for evaluating renal function and validating the clinical significance of these genes as potential biomarkers. Despite screening potential immune-related differentially expressed genes *via* bioinformatics analysis and preliminarily validating them in a mouse model using RT-PCR, this study has limitations. Currently, verification is only at the mRNA level, yet mRNA expression doesn’t always mirror protein expression. For future work, we’ll employ Western blot and immunofluorescence staining to validate key gene expression at the protein level in human DKD renal tissues and mouse models, enhancing conclusion reliability. We also plan gene experiments in *in vitro* cell models. By applying CCK-8 proliferation, scratch, and Transwell invasion assays, we aim to explore gene functions in DKD-related fibrosis, inflammation, and immune regulation. Besides experimental validation, future research will collect more clinical DKD patient samples or conduct cohort validation in large-scale external datasets. This will help assess gene expression stability across different populations and their clinical value. Therefore, the analysis of gene expression in different renal cell types and the investigation of their correlations with renal function indicators need further research, preferably in a larger cohort of clinical patients, and exploring the functional roles of the identified biomarkers in more detail.

In summary, this study systematically integrates multi-dataset bioinformatics analysis with machine learning to identify ALB and FOS as central immune-related biomarkers in DKD which are regulating macrophage–T cell interactions and cytokine-driven inflammation. Validated through multi-dataset integration and experiments, these genes have diagnostic potential and suggest a role for dysregulated innate immunity in DKD progression. Their link to immune infiltration shows a connection between chronic inflammation and renal injury, offering targets for immunomodulatory therapies. Clinically, this work provides a molecular framework for stratifying high-risk patients and precision medicine. Future research should prioritize validating these targets across different cohorts and disease stages to translate computational insights into therapeutic advancements.

## Supplementary Material

Supplementary Table S2.docx

Supplementary Table S1.docx

Supplementary Table S3.docx

Supplement Figure S1.tif

Supplementary Table S4.docx
