# Supplementary material for: Diagnostic immune-related markers for diabetic kidney disease: a bioinformatics and machine learning approach
Source: Ren Fail. 2025 Jul 10;47(1):2525467. doi: 10.1080/0886022X.2025.2525467 (PMC12247103; doi:10.1080/0886022X.2025.2525467)
Supplement: Supplementary Table S2.docx [file IRNF_A_2525467_SM3574.docx]

| ID | Description | Set Size | Enrichment Score | NES | P value | p. adjust | Q values | Rank | Leading edge | Core enrichment |
| --- | --- | --- | --- | --- | --- | --- | --- | --- | --- | --- |
| CHIANG_LIVER_CANCER_SUBCLASS_CTNNB1_DN | CHIANG_LIVER_CANCER_SUBCLASS_CTNNB1_DN | 10 | 0.64091855 | 2.15200994 | 0.00221412 | 0.01328471 | 0.01165325 | 13 | tags=80%, list=33%, signal=72% | LUM/MOXD1/CCL21/COL6A3/C7/CCL19/COL1A2/VCAN |
| HP_ABNORMAL_CARDIOVASCULAR_SYSTEM_PHYSIOLOGY | HP_ABNORMAL_CARDIOVASCULAR_SYSTEM_PHYSIOLOGY | 10 | -0.5427495 | -1.72495 | 0.02222814 | 0.02222814 | NA | 7 | tags=50%, list=18%, signal=55% | HBA1/LOX/ALB/FOS/G6PC |

**Table S2.** The results of GSEA in the three datasets.
