# Supplementary material for: Diagnostic immune-related markers for diabetic kidney disease: a bioinformatics and machine learning approach
Source: Ren Fail. 2025 Jul 10;47(1):2525467. doi: 10.1080/0886022X.2025.2525467 (PMC12247103; doi:10.1080/0886022X.2025.2525467)
Supplement: Supplementary Table S1.docx [file IRNF_A_2525467_SM3573.docx]

| **ID** | **Primer Name** | **Sequence** | **Size** |
| --- | --- | --- | --- |
| 01 | S100A9(F) | CTGCATGAGAACAACCCACG | 20bp |
| 02 | S100A9(R) | AGCTGTCACATGGCTGACC | 19bp |
| 03 | FOS(F) | GGAGAATCCGAAGGGAACGG | 20bp |
| 04 | FOS(R) | GCAATCTCAGTCTGCAACGC | 20bp |
| 07 | ALB(F) | CTCAGGTGTCAACCCCAACT | 20bp |
| 08 | ALB(R) | CTTCCACACAAGGCAGTCTCT | 21bp |

**Table S1.** Primer sequences used in this study.
