# Supplementary material for: Diagnostic immune-related markers for diabetic kidney disease: a bioinformatics and machine learning approach
Source: Ren Fail. 2025 Jul 10;47(1):2525467. doi: 10.1080/0886022X.2025.2525467 (PMC12247103; doi:10.1080/0886022X.2025.2525467)
Supplement: Supplementary Table S3.docx [file IRNF_A_2525467_SM3572.docx]

**Table S3.** The results of GSVA in the three datasets.

| Gene_Sets | logFC | Ave Expr | t | P. Value | adj. P.Val | B | regulate |
| --- | --- | --- | --- | --- | --- | --- | --- |
| BIOCARTA_DICER_PATHWAY | 0.424590396304904 | -0.0210579515030245 | 3.95318478271269 | 0.00015084441301656 | 0.0487150005669841 | 0.813811247652153 | Up |
| CERIBELLI_PROMOTERS_INACTIVE_AND_BOUND_BY_NFY | 0.358040540540623 | 0.00733677497712641 | 2.91332660887412 | 0.00448423122959415 | 0.149284031287899 | -2.09875333903954 | Up |
| FARMER_BREAST_CANCER_CLUSTER_4 | 0.360268016928825 | -0.0540685528354191 | 4.05713952305781 | 0.000103737548332694 | 0.0487150005669841 | 1.13976509216105 | Up |
| HOLLERN_ADENOMYOEPITHELIAL_BREAST_TUMOR | 0.35263537725098 | -0.0165873449564707 | 4.77865408943235 | 6.62455496652926e-06 | 0.0205692431710733 | 3.54839334004605 | Up |
| MATZUK_LUTEAL_GENES | -0.335902974148775 | 0.00695364140918441 | -4.82423295735388 | 5.52185927516175e-06 | 0.0205692431710733 | 3.70837627682212 | Down |
| MATZUK_MATERNAL_EFFECT | -0.322734178750703 | 0.00241269051656126 | -3.81383151794409 | 0.00024683175234267 | 0.0589548146941531 | 0.386016422118016 | Down |
| REACTOME_ACYL_CHAIN_REMODELING_OF_DAG_AND_TAG | -0.32990377547576 | -0.00350671950680264 | -3.19105914784293 | 0.00193708878601704 | 0.119249634911063 | -1.38733537042052 | Down |
| REACTOME_ATTACHMENT_OF_GPI_ANCHOR_TO_UPAR | -0.319333880637531 | 0.0176685007079742 | -3.34209587291143 | 0.00120146870783534 | 0.110808608993379 | -0.979132788477206 | Down |
| REACTOME_COMPETING_ENDOGENOUS_RNAS_CERNAS_REGULATE_PTEN_TRANSLATION | 0.371255736869033 | -0.00925296082609308 | 3.09914580093416 | 0.00257187302977575 | 0.122856396268519 | -1.6284963213288 | Up |
| REACTOME_FIBRONECTIN_MATRIX_FORMATION | 0.345502807390245 | -0.00940594694527438 | 3.36897643963172 | 0.00110187807060899 | 0.109434508365541 | -0.904962207426671 | Up |
| REACTOME_FORMATION_OF_XYLULOSE_5_PHOSPHATE | -0.324197080387522 | 0.0193292353952774 | -2.89034267768698 | 0.004795666860114 | 0.151376759064554 | -2.15527674871233 | Down |
| REACTOME_FORMYL_PEPTIDE_RECEPTORS_BIND_FORMYL_PEPTIDES_AND_MANY_OTHER_LIGANDS | 0.441951365283311 | 0.00618597730700128 | 4.30731778864445 | 4.11505462802221e-05 | 0.0425908154000298 | 1.94701885013208 | Up |
| REACTOME_PHENYLALANINE_METABOLISM | -0.342350515602693 | 0.0149733033303985 | -3.0756975793425 | 0.00276228164475501 | 0.128013201596482 | -1.68912359604567 | Down |
| REACTOME_RUNX1_REGULATES_TRANSCRIPTION_OF_GENES_INVOLVED_IN_DIFFERENTIATION_OF_KERATINOCYTES | 0.451745613683744 | -0.0294758844771271 | 4.09885992453384 | 8.91180939500324e-05 | 0.0487150005669841 | 1.27217770050676 | Up |
| REACTOME_VLDL_ASSEMBLY | -0.343776209480462 | 0.0219740273583604 | -3.11586688555016 | 0.00244362093387305 | 0.122378112897997 | -1.58503893975495 | Down |
| STANHILL_HRAS_TRANSFROMATION_UP | -0.373495147462651 | -0.0315204053867765 | -3.78570248635747 | 0.000272265030721347 | 0.0626209570659099 | 0.300968284788257 | Down |
| WP_CEREBRAL_ORGANIC_ACIDURIAS_INCLUDING_DISEASES | -0.331442422290367 | 0.0113640772326657 | -2.95800749693018 | 0.00393145191991292 | 0.145323312039638 | -1.98782662551446 | Down |
| WP_EXRNA_MECHANISM_OF_ACTION_AND_BIOGENESIS | 0.424590396304904 | -0.0210579515030245 | 3.95318478271269 | 0.00015084441301656 | 0.0487150005669841 | 0.813811247652153 | Up |
| WP_MICRORNA_NETWORK_ASSOCIATED_WITH_CHRONIC_LYMPHOCYTIC_LEUKEMIA | 0.337214405675583 | -0.0264294492742047 | 3.46346011740158 | 0.000810007622720714 | 0.104794736189492 | -0.64068644587815 | Up |
| WP_TRANSSULFURATION_PATHWAY | -0.323914416457763 | 0.00600990595183111 | -3.6144973984158 | 0.000489674222417691 | 0.0805703809633776 | -0.206932507572938 | Down |
