# Supplementary figures and images for: Diagnostic immune-related markers for diabetic kidney disease: a bioinformatics and machine learning approach
Source: Ren Fail. 2025 Jul 10;47(1):2525467. doi: 10.1080/0886022X.2025.2525467 (PMC12247103; doi:10.1080/0886022X.2025.2525467)

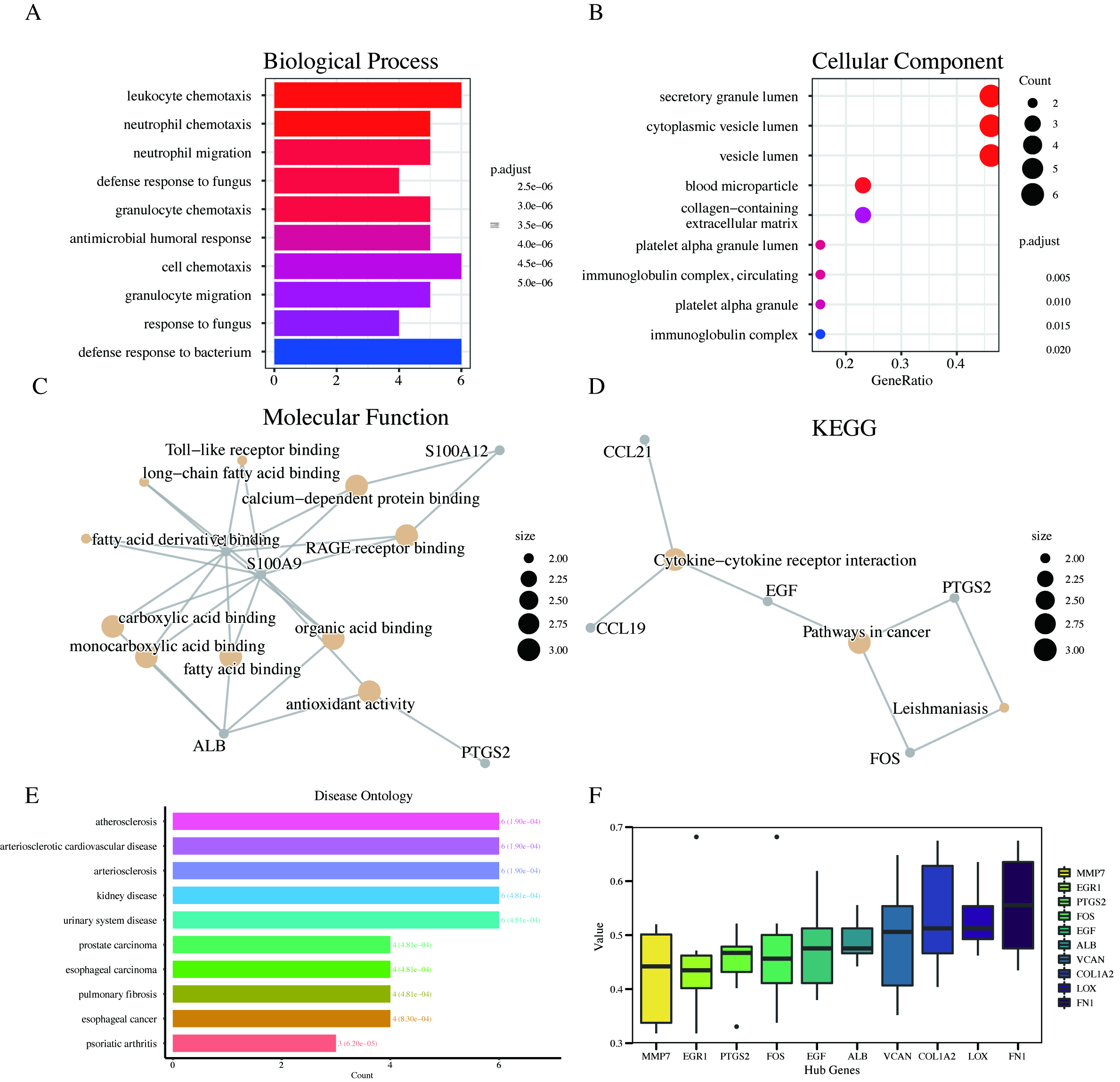

Supplement: Supplement Figure S1.tif [file IRNF_A_2525467_SM3571.tif]
