# Supplementary material for: Diagnostic immune-related markers for diabetic kidney disease: a bioinformatics and machine learning approach
Source: Ren Fail. 2025 Jul 10;47(1):2525467. doi: 10.1080/0886022X.2025.2525467 (PMC12247103; doi:10.1080/0886022X.2025.2525467)
Supplement: Supplementary Table S4.docx [file IRNF_A_2525467_SM3570.docx]

**Table S4.** DO, GO, and KEGG analyses results of DEGs (top 10 according to adjusted P-value).

| ONTOLOGY | ID | Description | Gene Ratio | Bg Ratio | P value | P.adjust | Q value | Gene ID | Count |
| --- | --- | --- | --- | --- | --- | --- | --- | --- | --- |
| BP | GO:0030595 | leukocyte chemotaxis | 6/13 | 232/18866 | 5.17651313323135e-09 | 2.37804826516942e-06 | 1.02717874248697e-06 | S100A12/S100A9/S100A8/CCL21/CCL19/DEFA1B | 6 |
| BP | GO:0030593 | neutrophil chemotaxis | 5/13 | 103/18866 | 5.46677762107913e-09 | 2.37804826516942e-06 | 1.02717874248697e-06 | S100A12/S100A9/S100A8/CCL21/CCL19 | 5 |
| BP | GO:1990266 | neutrophil migration | 5/13 | 122/18866 | 1.28577405434707e-08 | 2.73904180124097e-06 | 1.18310698492804e-06 | S100A12/S100A9/S100A8/CCL21/CCL19 | 5 |
| BP | GO:0050832 | defense response to fungus | 4/13 | 41/18866 | 1.35294079654779e-08 | 2.73904180124097e-06 | 1.18310698492804e-06 | S100A12/S100A9/S100A8/DEFA1B | 4 |
| BP | GO:0071621 | granulocyte chemotaxis | 5/13 | 127/18866 | 1.57416195473619e-08 | 2.73904180124097e-06 | 1.18310698492804e-06 | S100A12/S100A9/S100A8/CCL21/CCL19 | 5 |
| BP | GO:0019730 | antimicrobial humoral response | 5/13 | 137/18866 | 2.30486662455487e-08 | 3.34205660560455e-06 | 1.44357435958963e-06 | S100A12/S100A9/S100A8/IGHA1/DEFA1B | 5 |
| BP | GO:0060326 | cell chemotaxis | 6/13 | 311/18866 | 2.97763726641276e-08 | 3.70077774539871e-06 | 1.59852105881106e-06 | S100A12/S100A9/S100A8/CCL21/CCL19/DEFA1B | 6 |
| BP | GO:0097530 | granulocyte migration | 5/13 | 150/18866 | 3.63328819788539e-08 | 3.95120091520037e-06 | 1.70668932453301e-06 | S100A12/S100A9/S100A8/CCL21/CCL19 | 5 |
| BP | GO:0009620 | response to fungus | 4/13 | 54/18866 | 4.20408739074288e-08 | 4.06395114438479e-06 | 1.75539087543299e-06 | S100A12/S100A9/S100A8/DEFA1B | 4 |
| BP | GO:0042742 | defense response to bacterium | 6/13 | 348/18866 | 5.80574574541184e-08 | 5.0509987985083e-06 | 2.18173813801266e-06 | S100A12/S100A9/S100A8/IGHG4/IGHA1/DEFA1B | 6 |
| CC | GO:0034774 | secretory granule lumen | 6/13 | 322/19559 | 2.9592636956283e-08 | 4.18329573026159e-07 | 2.89701920378227e-07 | S100A12/S100A9/ALB/S100A8/EGF/DEFA1B | 6 |
| CC | GO:0060205 | cytoplasmic vesicle lumen | 6/13 | 326/19559 | 3.18467717306875e-08 | 4.18329573026159e-07 | 2.89701920378227e-07 | S100A12/S100A9/ALB/S100A8/EGF/DEFA1B | 6 |
| CC | GO:0031983 | vesicle lumen | 6/13 | 328/19559 | 3.30260189231178e-08 | 4.18329573026159e-07 | 2.89701920378227e-07 | S100A12/S100A9/ALB/S100A8/EGF/DEFA1B | 6 |
| CC | GO:0072562 | blood microparticle | 3/13 | 148/19559 | 0.000114853103951433 | 0.00109110448753861 | 0.000755612525996268 | ALB/IGHG4/IGHA1 | 3 |
| CC | GO:0031093 | platelet alpha granule lumen | 2/13 | 67/19559 | 0.000879950173021366 | 0.00668762131496238 | 0.00463131670011245 | ALB/EGF | 2 |
| CC | GO:0042571 | immunoglobulin complex, circulating | 2/13 | 76/19559 | 0.00113044433968107 | 0.00715948081798014 | 0.00495808920912752 | IGHG4/IGHA1 | 2 |
| CC | GO:0031091 | platelet alpha granule | 2/13 | 91/19559 | 0.00161516467931408 | 0.00876803683056216 | 0.00607204766659429 | ALB/EGF | 2 |
| CC | GO:0062023 | collagen-containing extracellular matrix | 3/13 | 427/19559 | 0.00251052475998149 | 0.0119249926099121 | 0.00825830513151807 | S100A9/S100A8/DEFA1B | 3 |
| CC | GO:0019814 | immunoglobulin complex | 2/13 | 163/19559 | 0.00506899149560976 | 0.021402408537019 | 0.0148216125602625 | IGHG4/IGHA1 | 2 |
| MF | GO:0050786 | RAGE receptor binding | 3/13 | 11/18352 | 4.5666900691625e-08 | 2.83134784288075e-06 | 1.05754927917447e-06 | S100A12/S100A9/S100A8 | 3 |
| MF | GO:0005504 | fatty acid binding | 3/13 | 39/18352 | 2.50059360346559e-06 | 7.75184017074334e-05 | 2.89542417243384e-05 | S100A9/ALB/S100A8 | 3 |
| MF | GO:0033293 | monocarboxylic acid binding | 3/13 | 72/18352 | 1.6099706707078e-05 | 0.000314651612417743 | 0.00011752691805077 | S100A9/ALB/S100A8 | 3 |
| MF | GO:0048306 | calcium-dependent protein binding | 3/13 | 85/18352 | 2.65212767387388e-05 | 0.000314651612417743 | 0.00011752691805077 | S100A12/S100A9/S100A8 | 3 |
| MF | GO:0016209 | antioxidant activity | 3/13 | 86/18352 | 2.74686300562698e-05 | 0.000314651612417743 | 0.00011752691805077 | S100A9/ALB/PTGS2 | 3 |
| MF | GO:0035325 | Toll-like receptor binding | 2/13 | 12/18352 | 3.04501560404267e-05 | 0.000314651612417743 | 0.00011752691805077 | S100A9/S100A8 | 2 |
| MF | GO:0036041 | long-chain fatty acid binding | 2/13 | 15/18352 | 4.83853798026089e-05 | 0.000428556221108822 | 0.000160071933181563 | S100A9/S100A8 | 2 |
| MF | GO:1901567 | fatty acid derivative binding | 2/13 | 29/18352 | 0.000186046283787524 | 0.00144185869935331 | 0.000538555032016518 | S100A9/S100A8 | 2 |
| MF | GO:0031406 | carboxylic acid binding | 3/13 | 212/18352 | 0.000399075015646457 | 0.00274918344112004 | 0.0010268596893827 | S100A9/ALB/S100A8 | 3 |
| MF | GO:0043177 | organic acid binding | 3/13 | 224/18352 | 0.000468797119767437 | 0.00276211664709309 | 0.00103169042845582 | S100A9/ALB/S100A8 | 3 |
| DO | DOID:9008 | psoriatic arthritis | 3/11 | 10/8007 | 2.30297693029732e-07 | 6.1950079424998e-05 | 3.07871652787116e-05 | S100A12/S100A9/S100A8 | 3 |
| DO | DOID:1936 | atherosclerosis | 6/11 | 344/8007 | 2.31724521764203e-06 | 0.000190444929366793 | 9.46449071789582e-05 | S100A12/S100A9/ALB/PTGS2/S100A8/EGF | 6 |
| DO | DOID:2348 | arteriosclerotic cardiovascular disease | 6/11 | 345/8007 | 2.35695206052171e-06 | 0.000190444929366793 | 9.46449071789582e-05 | S100A12/S100A9/ALB/PTGS2/S100A8/EGF | 6 |
| DO | DOID:2349 | arteriosclerosis | 6/11 | 356/8007 | 2.83189486047276e-06 | 0.000190444929366793 | 9.46449071789582e-05 | S100A12/S100A9/ALB/PTGS2/S100A8/EGF | 6 |
| KEGG | hsa04060 | Cytokine-cytokine receptor interaction | 3/5 | 265/5894 | 0.000840159707136546 | 0.0145257511491849 | 0.0103755365351321 | CCL21/EGF/CCL19 | 3 |
| KEGG | hsa05140 | Leishmaniasis | 2/5 | 73/5894 | 0.00147709750034009 | 0.0145257511491849 | 0.0103755365351321 | FOS/PTGS2 | 2 |
| KEGG | hsa05200 | Pathways in cancer | 3/5 | 327/5894 | 0.00155633048026981 | 0.0145257511491849 | 0.0103755365351321 | FOS/PTGS2/EGF | 3 |

*‘‘Count’’ means how many DEGs are involved.*
